# Analysis of Expression Pattern of snoRNAs in Human Cells A549 Infected by Influenza A Virus

**DOI:** 10.3390/ijms232213666

**Published:** 2022-11-08

**Authors:** Evgenii Zhuravlev, Mariia Sergeeva, Sergey Malanin, Rinat Amirkhanov, Dmitriy Semenov, Tatiana Grigoryeva, Andrey Komissarov, Grigory Stepanov

**Affiliations:** 1 Institute of Chemical Biology and Fundamental Medicine, Siberian Branch of the Russian Academy of Sciences, Novosibirsk 630090, Russia; 2 Smorodintsev Research Institute of Influenza, Ministry of Health of the Russian Federation, St. Petersburg 197376, Russia; 3 Institute of Fundamental Medicine and Biology, Kazan Federal University, Kazan 420008, Russia

**Keywords:** ncRNAs, snoRNAs, sdRNAs, influenza A virus, H1N1, RNA-seq, human cells, A549, transcriptome

## Abstract

Small nucleolar RNAs (snoRNAs) are a highly expressed class of non-coding RNAs known for their role in guiding post-transcriptional modifications of ribosomal RNAs and small nuclear RNAs. Emerging studies suggest that snoRNAs are also implicated in regulating other vital cellular processes, such as pre-mRNA splicing and 3′-processing of mRNAs, and in the development of cancer and viral infections. There is an emerging body of evidence for specific snoRNA’s involvement in the optimal replication of RNA viruses. In order to investigate the expression pattern of snoRNAs during influenza A viral infection, we performed RNA sequencing analysis of the A549 human cell line infected by influenza virus A/Puerto Rico/8/1934 (H1N1). We identified 66 that were upregulated and 55 that were downregulated in response to influenza A virus infection. The increased expression of most C/D-box snoRNAs was associated with elevated levels of 5’- and 3’-short RNAs derived from this snoRNA. Analysis of the poly(A)+ RNA sequencing data indicated that most of the differentially expressed snoRNAs synthesis was not correlated with the corresponding host genes expression. Furthermore, influenza A viral infection led to an imbalance in the expression of genes responsible for C/D small nucleolar ribonucleoprotein particles’ biogenesis. In summary, our results indicate that the expression pattern of snoRNAs in A549 cells is significantly altered during influenza A viral infection.

## 1. Introduction

Influenza viruses are enveloped single-stranded segmented negative-sense RNA viruses of the Orthomyxoviridae family, which are classified into four genera: influenza virus A–D (IAV, IBV, ICV, and IDV) [1]. The greatest risk to human health is associated with two types of influenza virus: A and B. Influenza A virus is the cause of annual seasonal epidemics and global pandemics, such as Spanish flu in 1918 and swine flu in 2009 [2,3]. At the cellular level, influenza A virus infection activates host viral RNA sensors (RIG-I, MDA5, TLRs), which induce downstream signaling pathways and regulate the expression pattern of antiviral genes [4]. It was also shown that the expression and function of long non-coding RNAs (lncRNAs) and microRNAs (miRNAs) are dysregulated by influenza virus infection [5,6]. However, the effects of this process on other non-coding RNAs (ncRNAs), including small nucleolar RNAs (snoRNAs), are less understood.

Small nucleolar RNAs are a class of small ncRNA 60–300 nt long that are widely present in eukaryotic cells’ nucleolus. In the human genome, snoRNAs are predominantly encoded by intronic regions of both non-coding and protein-coding genes [7]. It is generally accepted that snoRNAs act as guide molecules for functional small nucleolar ribonucleoprotein particles (snoRNPs) in the post-transcriptional processing of ribosomal RNA (rRNA) and small nuclear RNA (snRNA). There are two main groups of snoRNAs: C/D-box snoRNAs, which are associated with 2′-O-methylation, and H/ACA-box snoRNAs, which are associated with pseudouridylation [8].

However, recent studies have indicated that some snoRNAs are expressed at different levels with regard to the cell type and biological and environmental factors [9,10,11]. Several snoRNAs possess non-canonical functions, for instance, they guide cytosine acetylation and regulate alternative splicing and 3′-processing of mRNAs [12,13,14,15]. Additionally, high-throughput sequencing has revealed that snoRNAs can also be processed to generate smaller fragments called sno-derived RNAs (sdRNAs), which have important functional significance [16,17]. Some sdRNAs mainly derived from H/ACA-box snoRNAs (H/ACA-sdRNAs) play miRNA-like roles, interacting with Dicer and AGO complexes, whereas sdRNAs mainly derived from C/D-box snoRNAs (C/D-sdRNAs) exert alternative functions by binding other specific proteins or RNAs [18,19,20,21].

Moreover, the accumulated knowledge indicates that the expression of snoRNAs and sdRNAs is often changed in pathological conditions such as cancer or viral infection [22,23,24,25]. Several snoRNAs have been proposed as candidates for oncogene or tumor suppressor genes. The expression of short microRNA-like sdRNA-93 derived from SNORD93 contributed to the malignant phenotype of breast cancer [26]. SNORD89 overexpression promoted the proliferation and migration of endometrial cancer cells and inhibited apoptosis by downregulating the tumor suppressor gene BIM [27]. SNORD47 acted as a tumor suppressor, inhibiting the proliferation of glioma cells and inducing G2 phase arrest [28]. There is also growing evidence for functional interactions between snoRNAs and viruses [25]. Knock-down studies revealed that specific H/ACA-box and C/D-box snoRNAs are involved in virus–host interactions and virus-induced cell death [29]. Moreover, retroviruses package ncRNAs, including snoRNAs, into their virions [30,31]. Several nuclear and cytosolic RNA viruses use a short capped snoRNA fragment to prime viral mRNA transcription (‘cap-snatching’ strategy) [32,33]. Despite these reports, many questions on how snoRNAs interact with pathogens at a molecular level and whether regulation of snoRNAs and sdRNAs is an inductor or an indicator in disease progression remain open.

Here, we performed small RNA sequencing analysis of influenza-A-virus-infected A549 cells, which revealed that 121 snoRNAs show a significant change in their expression in response to infection. In addition, we showed an association between upregulated expression of most C/D-box snoRNAs and an increase in the level of 5′- and 3′-short RNA derived from these snoRNAs. Based on the parallel analysis of poly (A)+ RNA sequencing data, we examined the correlation between the levels of intronic C/D-box snoRNA and corresponding host genes and the change in the expression of genes responsible for C/D snoRNP biogenesis during influenza A viral infection.

## 2. Results

### 2.1. Library Preparation: Sequencing and Mapping

**Figure 1:**
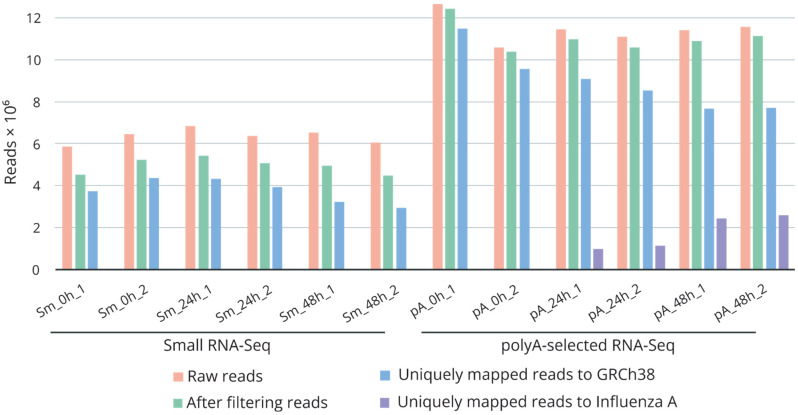
Summary of RNA sequencing data. Number of retrieved raw reads, reads after filtering (trimming of adapters, filtering by quality, removal of ribosomal RNA fragments), and uniquely mapped reads to GRCh38 and to the influenza A/Puerto Rico/8/1934 genomes. Sm_0h_1/2, Sm_24h_1/2, Sm_48h_1/2—small RNA libraries; pA_0h_1/2, pA_24h_1/2, pA_48h_1/2—poly(A)+ RNA libraries. 0h—non-infected cells; 24 and 48h—influenza-A/Puerto Rico/8/1934-infected cells after 24 and 48 h of incubation.

For systematic investigation of small nucleolar RNAs’ regulation during influenza A viral infection, we performed RNA sequencing analysis of A/Puerto Rico/8/1934-virus-infected A549 cells. We generated six small RNA and six poly(A) + RNA libraries using two biological replicates of each time point: before infection (0 h) and after infection (24 and 48 h). The sequencing reactions yielded approximately 6.4 and 11.5 million raw reads for the small RNA and poly(A)+ RNA libraries, respectively (Figure 1, Table S2). Raw reads were processed and aligned via STAR to a combined genomic reference of the human genome and the genome of influenza A virus. The percentage of uniquely mapped reads ranged from 50% to 68% and from 87% to 91% for the small RNA and poly(A)+ RNA libraries, respectively. Extension of the infected cells’ incubation time correlated with an increase in the number of reads mapped to the influenza A virus genome. The majority of the annotated reads of the small RNA libraries were mapped to pre-miRNAs (59.6 to 62.5%) and snoRNAs (35.5 to 39.2%) (Figure S3A).

### 2.2. Differentially Expressed Small Nucleolar RNAs in Response to Influenza A Virus Infection

**Figure 2:**
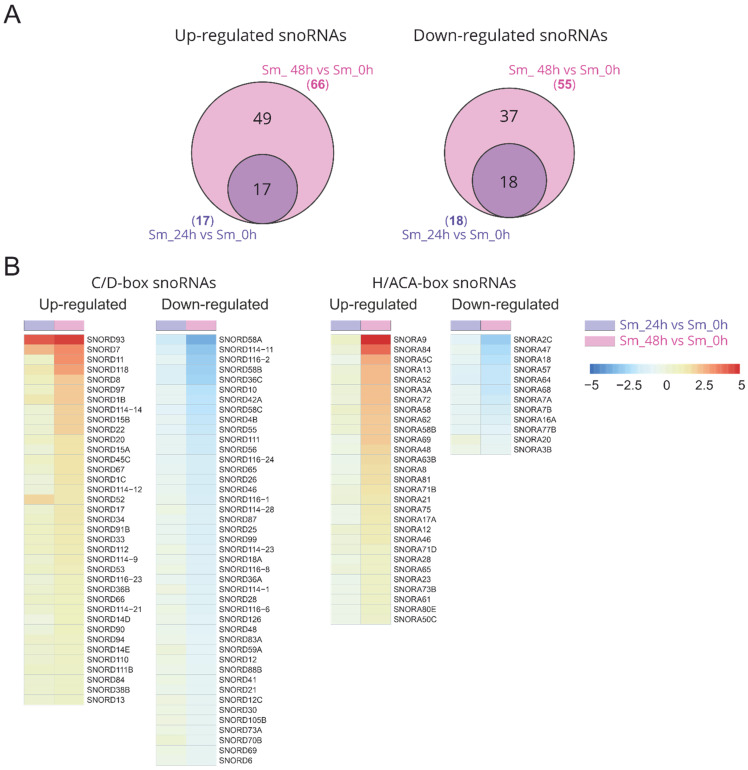
snoRNAs that are differentially expressed during virus infection. (**A**) Venn diagrams show the number of common and unique upregulated and downregulated snoRNAs 24 and 48 h after influenza A virus infection (FDR-adjusted *p*-value < 0.05, absolute value of a log2 (FC) > 0.58). (**B**) Overview of 121 differentially expressed snoRNAs in A549 cells during influenza A virus infection. Colors on the heat map indicate the log2 ratios of the expression level in virus-infected cells relative to the expression level in non-infected cells (normalized read counts). Red, upregulation; blue, downregulation. Sm_24h vs. Sm_0h, infected cells after 24 h of incubation vs. non-infected cells; Sm_48h vs. Sm_0h, infected cells after 48 h of incubation vs. non-infected cells.

Differential analysis of the small RNA expression revealed that large numbers of snoRNAs were upregulated or downregulated in response to influenza A virus infection (Figure S2). An increase in the incubation time of the infected cells led to an increase in both the level of changes and the number of unique differentially expressed C/D-box and H/ACA-box snoRNAs (SNORDs and SNORAs) (Figure 2, Table S3). Then, 48 h after infection, 38 and 43 C/D-box snoRNAs and 29 and 13 H/ACA-box snoRNAs were upregulated and downregulated, respectively. The number of differentially expressed snoRNAs was even higher than the number of differentially expressed mature miRNAs (Figure S3B; Table S4).

### 2.3. Upregulated C/D-Box snoRNAs Are Actually Upregulated sno-Derived RNAs

C/D-box snoRNAs were examined in more detail. Firstly, 99% of all reads mapped to snoRNAs were aligned to C/D-box snoRNAs (on average, 1.17 × 10^6^ reads to C/D-box vs. 7.73 × 10³ reads to H/ACA-box for each sample). Secondly, the selected sequencing protocol (75-nucleotide single-end reads) is more suitable for the analysis of the C/D-box snoRNA distribution (an average length is 60–90 nucleotides) then for the analysis of H/ACA-box snoRNAs (an average length is about 150 nucleotides). Thirdly, we focused on the C/D-box snoRNAs as more structure and function diversity has, up to now, been identified among this subclass compared with H/ACA-box [34]. Furthermore, there is an emerging body of evidence for functional interactions between C/D box snoRNAs and RNA viruses [25].

**Figure 3:**
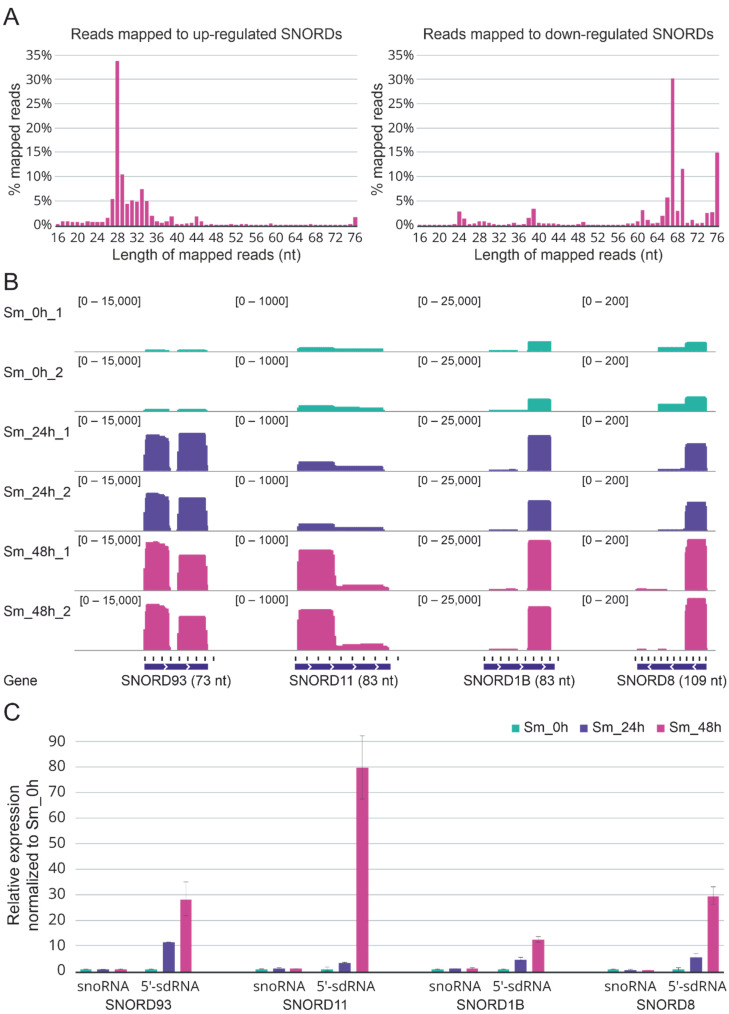
(**A**) Size distribution of reads mapped to upregulated and downregulated C/D-box snoRNAs (SNORDs) in infected cells after 48 h of incubation. (**B**) The coverage tracks of the aligned reads for the four snoRNAs that were upregulated in response to influenza A virus infection (SNORD93, SNORD11, SNORD1B, and SNORD8) generated with IGV. Green reads, non-infected cells; violet reads, infected cells after 24 h of incubation; pink reads, infected cells after 48 h of incubation. (**C**) Expression of full-length SNORD93, SNORD11, SNORD1B, and SNORD8 and their 5′-sdRNAs measured by quantitative RT-PCR with custom stem-loop primers.

We examined the match length distribution for experimental reads mapped to the reference sequence of upregulated and downregulated C/D-box snoRNAs genes. Interestingly, most reads that mapped to upregulated C/D-box snoRNAs had a size distribution between 26 and 35 nt while most reads mapped to downregulated C/D-box snoRNAs had a size distribution between 65 and 76 nt, more specifically for C/D-box snoRNAs. The A549 cells at 48 h post infection were characterized by the most pronounced effect (Figure 3A). A similar pattern in the case of upregulated and downregulated H/ACA-box snoRNAs was also observed. Furthermore, elongation of the infected cells’ incubation time resulted in a redistribution of the forms of upregulated C/D-box snoRNAs from mature RNAs to processing fragments of the mature RNAs (Figure S4).

The reads aligned to C/D-box snoRNAs were also visualized using the Integrative Genomics Viewer (IGV) [35]. Visual analysis of the mapped reads revealed once again that while a decrease in the full-length forms was typical for downregulated C/D-box snoRNAs, an increase in 5′- and 3′-short RNA derived from snoRNAs was common to upregulated C/D-box snoRNAs (Figure S5A; Figure 3B). Importantly, these short snoRNAs fragments did not likely represent the degradation products as fixed excision patterns were characterized for most of them.

To validate our sequencing data, we tested the expression of upregulated SNORD93, SNORD11, SNORD1B, SNORD8, and derived sdRNAs in independent samples of A549 cells infected with influenza A/Puerto Rico/8/1934 virus by real-time quantitative reverse transcription PCR (RT-PCR) with custom stem-loop primers [36]. The RT-PCR analysis confirmed the processing patterns for 5′-sdRNAs of the selected C/D-box snoRNAs and the influenza-associated upregulation of the corresponding sdRNAs observed in the sequencing data (Figure 3C). Furthermore, we also verified the influenza-associated decreased expression of some downregulated C/D-box snoRNAs. The results for SNORD58A, SNORD42A, and SNORD79 are presented in Figure S5B.

### 2.4. C/D-Box snoRNA Host Genes and C/D snoRNP Biogenesis during Influenza A Viral Infection

**Figure 4:**
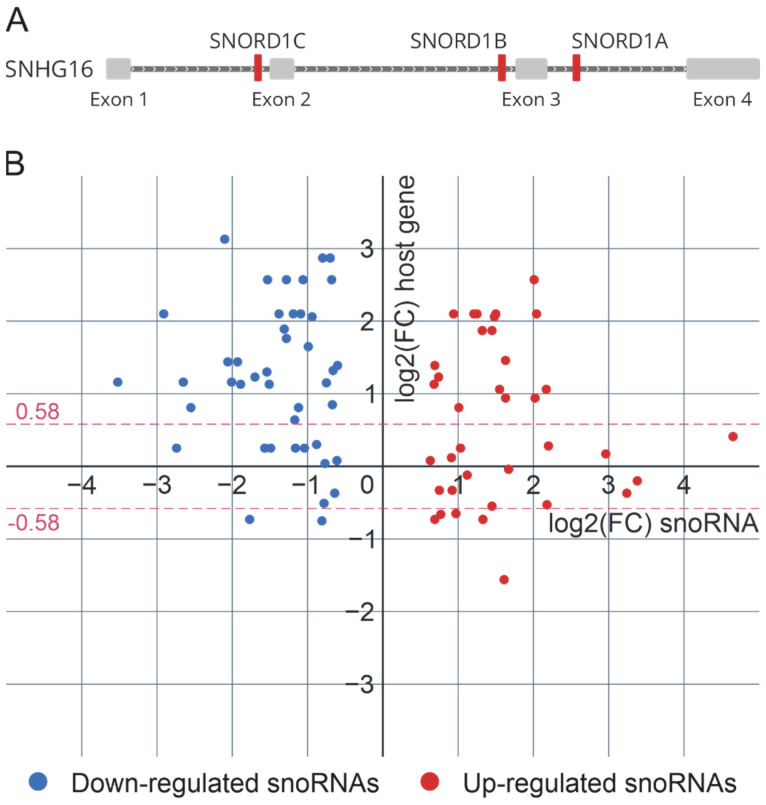
(**A**) Intronic organization of snoRNA genes located in small nucleolar RNA host gene 16 (*SNHG16*) (RefSeq: NR_038108.1). (**B**) Relative expression levels of differentially expressed snoRNAs and corresponding host genes. Each dot represents one intronic snoRNA. The position on the *x*-axis represents its relative expression in virus-infected cells (48 h after infection) vs. non-infected cells (small RNA sequencing), and the position on the *y*-axis represents the relative expression of the corresponding host gene (poly(A)+ RNA sequencing). The data sets with identical y-values correspond to snoRNAs encoded in the same host gene.

In the human genome, most C/D-box snoRNA genes are located within the introns of host genes encoding mRNA or long noncoding RNA (Figure 4A). The biogenesis of such C/D-box snoRNAs and the assembly of snoRNPs are related to the splicing machinery and require a number of trans-acting factors. Therefore, using the data of the differential analysis of the expression of poly(A)+ RNAs (virus-infected cells vs. non-infected cells), we examined the correlation between the intronic snoRNA genes’ expression and the expression of corresponding host genes. Moreover, we looked at the changes in the expression of genes encoding proteins involved in C/D snoRNP biogenesis during influenza A virus infection.

According to our data, the synthesis of most differentially expressed snoRNAs is not correlated with the expression of the host genes. Only 25% of all differentially expressed C/D-box snoRNAs (18 upregulated and 2 downregulated snoRNAs) and corresponding host genes were characterized by significant unidirectional changes 48 h after influenza A virus infection (Figure 4B). To note, some host genes (*MIG8*, *RPL7A*, *SNHG1*, *SNHG14*, *SNHG32*) contain both upregulated and downregulated snoRNAs located in different introns. Recent studies have shown that the expression of individual snoRNAs and their cognate spliced RNA can be uncoupled via alternative splicing and nonsense-mediated decay [37].

The differential expression analysis of genes responsible for C/D snoRNP biogenesis revealed an imbalance in the mRNA level of genes involved in the major steps of snoRNA maturation during influenza A viral infection (Table 1). While the mRNA levels of the core proteins SNU13 and NOP56 increased, the mRNA level of the assembly factors RPAP3, RUVBL1, and RUVBL2 decreased. Moreover, the mRNA levels of the transcription factor MYC, known as the master regulator of snoRNP biogenesis, and the general splicing factor AQR, which binds the splicing and snoRNP biogenesis machineries, also changed in virus-infected cells compared to non-infected cells.

### 2.5. Modulation of rRNA 2′-O-methylation in Response to Influenza A Virus Infection

The main function of C/D-box snoRNAs is to guide site-specific 2′-O-methylation of rRNA nucleotides [38]. The rRNA modification profile plays an important role in global ribosome topology and functions [39]. According to an interactive database of human snoRNA snoDB, approximately 80% of detected differentially expressed snoRNAs contain antisense boxes that are complementary to specific target rRNA nucleotides [40,41]. Additionally, it is worth noting that in both the case of downregulated and upregulated snoRNAs, we observed a decrease in the level of full-length forms of snoRNAs required for rRNA modification.

**Figure 5:**
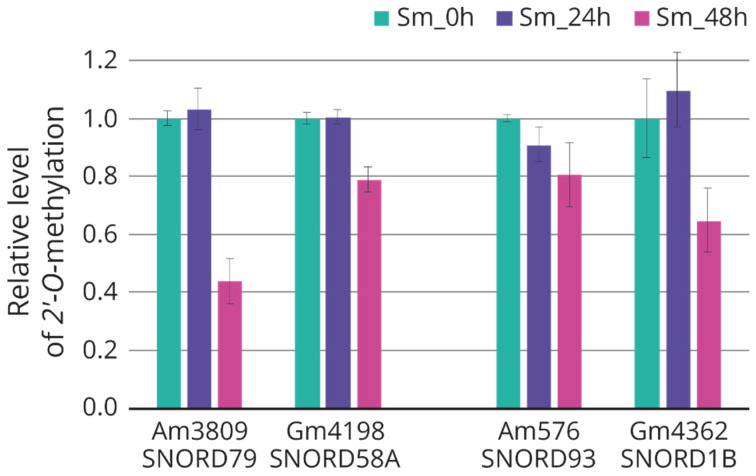
Relative level of rRNA 2’-O-methylation in A549 cells 24 and 48 h after influenza A virus infection. C/D-box snoRNA—target rRNA nucleotide: SNORD79—28S:Am3809; SNORD58a—28S:Gm4198; SNORD93—18S:Am576; SNORD1b—28S:Gm4362. Green, non-infected cells; violet, infected cells after 24 h of incubation; pink, infected cells after 48 h of incubation.

Using the method based on the termination of reverse transcription, we analyzed the changes in the target nucleotides of 2′-O-methylation for some differentially expressed snoRNAs (downregulated: SNORD79, SNORD58a; upregulated: SNORD93, SNORD1b). All selected nucleotides were characterized by a trend toward decreased 2′-O-methylation after 48 h of incubation after influenza A virus infection (Figure 5).

## 3. Discussion

Influenza A virus (IAV), causing annual seasonal epidemics and global pandemics, is of high public health concern. During the process of IAV infection, disturbances are observed at the different levels of gene expression, including activation of antiviral gene transcription, regulation of alternative splicing, changes in post-transcriptional modifications, and control of the translational machinery [42,43,44,45,46]. In recent years, it has been shown that host ncRNAs also play an important role in the regulation of influenza virus replication [5,6]. Some inhibit the replication and expression of the viral genome while others promote the initiation of the infection and escape from the host antiviral innate immune response.

In this paper, through RNA sequencing analysis of A549 human cells infected with influenza A/Puerto Rico/8/1934 (H1N1) virus, we found that a large number of snoRNAs are differentially expressed in human cells during influenza infection. Further, 48 h after infection, 38 and 43 C/D-box snoRNAs and 29 and 13 H/ACA-box snoRNAs were upregulated and downregulated, respectively. The main function of snoRNAs is known to guide site-specific rRNA modifications required for ribosome biogenesis [38,47]. Recent studies have shown that alterations in the rRNA modification profile may regulate and adapt the ribosome function in response to environmental changes, and in various diseases [48]. Furthermore, the synthesis of viral proteins and viral replication also depend on the host ribosome [49]. According to the experimental data, most of the detected differentially expressed snoRNAs have a known rRNA nucleotide as a target for 2′-O-methylation [40]. Interestingly, a decrease in the full-length forms was also observed in the case of upregulated snoRNAs. The analysis of the rRNA modifications revealed a trend toward decreased 2′-O-methylation of specific target rRNA nucleotides after 48 h of incubation after influenza A virus infection. It can therefore be assumed that specific regulation of snoRNA expression in response to influenza virus infection is aimed at changes in the rRNA modification pattern and adaptation of the translation apparatus. Such a type of control may contribute to the efficient translation of viral mRNAs and at the same time the suppression of cellular mRNA translation [42].

At present, it is known that the majority of ncRNA expression is dysregulated in response to influenza virus infection [5,6]. However, the number of studies focused on the regulation of snoRNA expression in infected cells is very limited. Earlier, the analysis of deep sequencing of the mouse lung transcriptome revealed 30 small RNAs overlapped with annotated snoRNAs, which were differentially expressed in mice during SARS-CoV and influenza A virus infection [50]. A recent study utilizing gene-trap insertional mutagenesis to randomly inactivate cellular genes found that 83 SNORA and SNORD genes are potentially important for viral replication. The silencing of SNORA/Ds encoded within RPS11 (SNORD35B), SNHG3 (SNORA73A, SNORA73B), and SNHG1 (SNORD22, SNORD25, SNORD26, SNORD27, SNORD28, SNORD29, SNORD30, SNORD31) inhibited infection with influenza A virus [29]. Interestingly, in our data, we observed that while the relative expression level of SNORD22 increased 4-fold, the level of SNORD26 and SNORD28 decreased 2-3-fold in influenza-A-virus-infected cells compared to non-infected cells. The observed changes in the expression of SNORDs indicate that some snoRNAs required for viral replication may be partially suppressed in the infected cells. Such regulation may be a component of the antiviral response. In addition, according to our data, the independently transcribed SNORD13 and SNORD118 were upregulated after influenza A virus infection. This may be due to the ability of the influenza A virus to use short capped snoRNA fragments to prime viral mRNA transcription (‘cap-snatching’ strategy), as previously shown by RNA sequencing [32,33].

Visual and size distribution analyses of the mapped reads revealed that upregulated C/D-box snoRNAs were associated with the increase in the level of 5′- and 3′-short RNA derived from snoRNAs. Such fragments of snoRNA processing were discovered in the late 2000s and were named sno-derived RNAs [16,17]. Next-generation sequencing technology has revealed that some sdRNAs are involved in gene expression regulation, and increased production of sdRNAs is frequently associated with the response to stress and development of various pathologies [51]. However, the mechanisms of the generation of smaller fragments from full-length snoRNAs remain poorly understood.

Despite the fact that most RNA fragments derived from C/D-box snoRNAs are different in size from miRNAs (17–19 nt or > 27 nt for snoRNAs vs. 21–22 nt for miRNAs) and are not efficiently incorporated into the Ago2 protein, a small number of sno-RNA-derived RNAs may carry out miRNA-like functions [18,52]. sdRNA-93 derived from SNORD93 could regulate the expression of the *PIPOX* gene through miRNA-like silencing, contributing to the malignant phenotype of breast cancer [26]. sdRNA-28 derived from SNORD28 could serve an miRNA-like role by changing the p53 protein stability via direct regulation of the *TAF9B* gene [53]. According to our data, SNORD93 (one of the most upregulated snoRNAs) and SNORD28 (the level of full-length SNORD28 was decreased) were also characterized by an increase in sdRNAs in response to influenza A virus infection.

It is worth noting that both previous studies and our study indicate that non-infected cells also contain similar fragments of snoRNA processing. Thus, influenza A virus infection merely influences the activity of the sdRNA formation process. It is known that viruses induce host shutoff when the cells begin to produce viral proteins at the expense of host proteins. The main cause for host shutoff during influenza A virus infection is selective degradation of host mRNAs by PA-X endonuclease. The analysis of the RNA-sequencing and ribosome profiling of A549 cells infected with A/Puerto Rico/8/1934 (H1N1) virus revealed a relative reduction in the RNA (2-4-fold) and translation (2-fold) levels of the main genes responsible for C/D-box snoRNA biogenesis: FBL, NOP56, NOP58, and NHP2L1 (SNU13), 12 h after infection [42]. In our study, we also observed an imbalance in the mRNA level of genes involved in the major steps of C/D-box snoRNA maturation, 24 and 48 h after infection (Table 1). We hypothesize that snoRNP core proteins can also be recruited by viral RNA by binding to secondary structure elements, in particular, K-turn in viral mRNAs [54,55]. A lack of core proteins of C/D snoRNPs may lead to deregulation of snoRNA biogenesis and the production sdRNAs. Moreover, the snoRNA-processing profiles suggest that sdRNAs arise from specific cleavage by ribonucleases at the 5′ and 3′ ends and protection from further processing by noncanonical co-transcriptionally associated proteins. Thus, it is possible that influenza A virus infection contributes to a switch between the functions performed by snoRNA or sdRNAs derived from it.

In conclusion, we demonstrated the expression pattern of snoRNAs in A549 human cells infected with influenza A virus. We also showed that some C/D-box snoRNAs are characterized by an increase in the sno-derived RNA level. The regulation of snoRNA biogenesis and processing during infection is yet another example of the intricate and poorly understood interaction between the host and virus. Further study of the interaction between snoRNAs and influenza viruses will likely uncover new functions of snoRNAs and possibly unveil new anti-viral strategies.

## 4. Materials and Methods

### 4.1. Virus and Cell Lines

A/Puerto Rico/8/1934 (H1N1) influenza virus from the collection of Smorodintsev Research Institute of Influenza (Ministry of Health of the Russian Federation) was used. A/Puerto Rico/8/1934 influenza virus were propagated in 10-day-old ECE. Virus titers were determined by estimating the 50% tissue culture infectious dose (TCID50) in MDCK cell culture (IRR #FR-58, Manassas, VA, USA). Virus stocks were stored at −80 °C until use.

A549 cells (ATCC #CCL-185, Manassas, VA, USA) were maintained in DMEM/F12 (Thermo Fisher Scientific, Waltham, MA, USA) supplemented with GlutaMAX (Thermo Fisher Scientific, Waltham, MA, USA) and 10% FBS (Thermo Fisher Scientific, Waltham, MA, USA) at 37 °C, 5% CO_2_.

### 4.2. Infection of Cells and Growth Kinetics of Influenza Virus

Cells were grown in T25 cell culture flasks (TPP, Trasadingen, Switzerland) until 90–100% monolayer. Influenza A/Puerto Rico/8/1934 virus was diluted in culture media (serum-free, 1% Antibiotic-Antimycotic (Thermo Fisher Scientific, Waltham, MA, USA), 1 μg/mL TPCK-trypsin (Merck, Darmstadt, Germany)) to reach a multiplicity of infection of 1 TCID50/cell. Cells monolayers were washed twice by DPBS (Thermo Fisher Scientific, Waltham, MA, USA) and subsequently virus inoculum was added (1 mL/flask) and incubated at 37 °C for 1 h, 5% CO_2_. As absorption passed, the inoculum was removed, and fresh media was added. Infected cells were incubated at 37 °C for 24 or 48 h, 5% CO_2_. At 24 and 48 h after infection, all the culture media were collected, and the titers were measured by the TCID50 assay. The growth kinetics of the influenza A/Puerto Rico/8/1934 (H1N1) virus in the cell lines are presented in Figure S1. Non-infected intact cells without incubation (time point 0 h) were included as controls. Following the end of the incubation period, the cells were washed twice with DPBS and directly lysed by the addition of phenol reagent LIRA (Biolabmix, Novosibirsk, Russia).

### 4.3. RNA Isolation

Total RNA and small RNA (< 200 nucleotide length) fractions were extracted from cells and were purified using the phenol-chloroform extraction method followed by isolation on absorption columns using the LRU-100–50 kit (Biolabmix, Novosibirsk, Russia) or analogical mirVana™ miRNA isolation kit (Thermo Scientific, Waltham, MA, USA) and diluted with nuclease-free water (Biolabmix, Novosibirsk, Russia). The RNA concentration was assessed using a Qubit RNA HS Assay Kit (Thermo Scientific, Waltham, MA, USA) with a Qubit 2 fluorometer (Thermo Fisher Scientific, Waltham, MA, USA). The quality of total RNA, expressed as the RNA integrity number (RIN), was determined with a Bioanalyzer 2100 instrument (Agilent, Santa Clara, CA, USA) using an Agilent RNA Pico 6000 Kit (Agilent, Santa Clara, CA, USA) [56]. A threshold RIN value greater than 7.0 was taken as the cut-off point for the transition to the stage of library preparation. The efficiency of enrichment for small RNA and their length size distribution were evaluated using 1.5% TAE-agarose gel (Thermo Fisher Scientific, Waltham, MA, USA) and a Bioanalyzer 2100 instrument with an Agilent Small RNA kit (Agilent, Santa Clara, CA, USA), respectively. For the sequencing libraries’ preparation and RT-qPCR analysis, solutions of the extracted total RNA and small RNA were treated with DNase I (Thermo Fisher Scientific, Waltham, MA, USA) to remove DNA.

### 4.4. Library Preparation and Sequencing

A total of 12 cDNA libraries (6 for small RNA and 6 for poly(A)+ RNA) were prepared from 2 biological replicates of each time point: before infection (0 h) and after infection (24 and 48 h). The construction of cDNA libraries was performed according to a standard protocol using a NEBNext Multiplex Small RNA Library Prep Kit for Illumina (New England Biolabs, Ipswich, MA, USA) for small RNA fraction, NEBNext Ultra II Directional RNA library preparation kit for Illumina (New England Biolabs, Ipswich, MA, USA), and NEBNext mRNA Magnetic Isolation Module (New England Biolabs, Ipswich, MA, USA) for poly(A)+ RNA fraction. For the prepared sequencing libraries, the fragment size distribution was analyzed using a Bioanalyzer 2100 instrument (Agilent, Santa Clara, CA, USA) with an Agilent High Sensitivity DNA Kit (Agilent, Santa Clara, CA, USA) and quantification by a Qubit DNA HS Assay Kit (Thermo Fisher Scientific, Waltham, MA, USA) with a Qubit 2 fluorometer (Thermo Fisher Scientific, Waltham, MA, USA). Libraries were sequenced on an Illumina NextSeq 500 instrument (Illumina, San Diego, CA, USA) in 75-base-pair-single-end mode (NextSeq 500/550 High Output v2.5 Kit (Illumina, San Diego, CA, USA)). Binary Base Call files provided by the Illumina Real-Time Analysis (RTA) software were de-multiplexed and converted into FASTQ format by bcl2fastq2 Conversion Software (v2.20, Illumina, San Diego, CA, USA). The construction of cDNA libraries and massive parallel sequencing were conducted at the Institute of Fundamental Medicine and Biology, Kazan Federal University (Kazan, Russia).

### 4.5. RNA-seq and Differential Expression Analysis

The raw data were saved as FASTQ format files. The quality control of the raw and trimmed reads was performed using FastQC (v0.11.9, Simon Andrews, Babraham, UK) and MultiQC (v1.9, Phil Ewels, Stockholm, Sweden) [57,58]. Trimming of the adapter content and quality trimming was performed using fastp (v0.21.0, Shifu Chen, Shenzhen, China) [59]. The reads complementary to ribosomal RNA were filtered out from the trimmed reads using SortMeRNA (v2.1b, Evguenia Kopylova, Villeneuve d’Ascq, France) [60]. The filtered reads were aligned via STAR (v2.7.7a, Alexander Dobin, Cold Spring Harbor, NY, USA) to a combined genomic reference of the human genome (GRCh38) and the genome of influenza A/Puerto Rico/8/1934 (H1N1) [61]. The counting of reads was performed with the featureCounts function from the R package Rsubread (v2.4.3, Yang Liao, Parkville, Australia) [62]. Differentially expressed RNAs were identified using R package DESeq2 (v1.30.1, Michael Love, Heidelberg, Germany) with an FDR-adjusted *p*-value < 0.05 and the absolute value of a log2 (FC) > 0.58 [63]. To detect differentially expressed mature miRNAs, trimmed reads of 18–25 base length were mapped to the human genome with Bowtie2 (v2.1.0, Ben Langmead, Baltimore, MD, USA) using the “very-sensitive-local” option [64,65]; reads matched to mature miRNAs from the miRBase database were counted by featureCounts [66]; the obtained counts were used for differential analysis by DESeq2. RNA-seq data were deposited in the ArrayExpress database at EMBL-EBI (https://www.ebi.ac.uk/biostudies/arrayexpress, accessed on 1 August 2022) under the accession number E-MTAB-12088 (small RNA fraction) and E-MTAB-9511 (poly(A)+ RNA fraction) [67].

### 4.6. RT-qPCR Analysis

RT-qPCR reaction was performed with BioMaster RT-qPCR SYBR Blue (Biolabmix, Novosibirsk, Russia), and two-step stem-loop RT-qPCR was performed with Reverse Transcriptase M-MuLV–RH (Biolabmix, Novosibirsk, Russia) and BioMaster UDG HS-qPCR (Biolabmix, Novosibirsk, Russia) using LightCycler 96 System (Roche, Basel, Switzerland). The primers employed in this study are presented in Table S1. The levels of snoRNAs or sdRNAs were represented as relative values normalized to the level of U6 and U11 small nuclear RNAs. Quantitative PCR data analysis was carried out using qbase+ software, version 3.1 (Biogazelle, Ghent, Belgium), which includes finding the stable reference genes with geNORM, quality control, and relative quantification of the snoRNA or sdRNA levels [68,69]. The mean values (±s.d.) for three biological samples were represented.

### 4.7. Analysis of the Relative Level of 2′-O-methylation of the Target rRNA Nucleotide

Reverse transcription followed by PCR with modification-specific primers was performed using total RNA samples. The primers employed for analysis of the methylation status of 28S:Am3809 (SNORD79); 28S:Gm4198 (SNORD58a); 18S:Am576 (SNORD93); and 28S:Gm4362 (SNORD1b) are presented in Table S1. Each primer set included a reverse primer “R” (located downstream of 2′-O-methylated nucleotide) and two overlapping forward primers: “F” (located upstream of 2′-O-methylated nucleotide) and “In” (covering 2′-O-methylated nucleotide). For each of the samples, RT reactions with 1.0 (optimal) and 3.0 mM (suboptimal) dNTP concentrations were performed in parallel. Subsequent qPCR reactions were performed with two pairs of primers: “F”/”R” and “In”/”R” for each RT point. The relative level of 2′-O-methylation was calculated with the 2^−∆∆Ct^ method, where ∆∆Ct = ∆Ct_3.0mM_ − ∆Ct_1.0mM_; ∆Ct = Ct_F/R_ − Ct_In/R_. The relative change in the modification level of the target nucleotide was evaluated based on the difference between the obtained values for the study (A549 cells 24 and 48 h after influenza A virus infection) and control (non-infected intact A549 cells) samples. This approach is based on the method of identification of the 2′-O-methylation groups in rRNA by RT-qPCR first presented by Belin et al. [70,71].

## Figures and Tables

**Table 1:** Differential expression of genes encoding proteins involved in eukaryotic C/D snoRNP biogenesis (FDR-adjusted *p*-value < 0.05, absolute value of a log2 (FC) > 0.58) [34].

Function	Ensembl ID	Gene Symbol	log2 (FC)	*p*.adj
Core proteins	ENSG00000105202	FBL	0.03	1.0 × 10^0^
	ENSG00000101361	NOP56	1.23	5.4 × 10^−46^
	ENSG00000055044	NOP58	−0.37	3.8 × 10^−4^
	ENSG00000100138	SNU13 (15.5 kDa)	0.96	3.4 × 10^−18^
Transcription factor	ENSG00000136997	MYC	1.01	6.1 × 10^−27^
General splicing factor	ENSG00000021776	AQR (IBP160)	−0.99	5.3 × 10^−19^
Formation of a tertiary complex with SNU13	ENSG00000083635	NUFIP1	0.43	1.1 × 10^−1^
	ENSG00000273611	ZNHIT3	0.59	1.1 × 10^−4^
Formation of the complex R2TP involved in stabilization and the recruitment of NOP58	ENSG00000096384	HSP90AB1	−0.29	2.0 × 10^−5^
	ENSG00000104872	PIH1D1	1.01	3.1 × 10^−16^
	ENSG00000005175	RPAP3	−1.01	2.4 × 10^−12^
	ENSG00000175792	RUVBL1	−1.37	1.1 × 10^−27^
	ENSG00000183207	RUVBL2	−1.91	1.7 × 10^−65^
Control of the nucleolar localization	ENSG00000166197	NOLC1 (NOPP140)	0.07	4.4 × 10^−1^
	ENSG00000164902	PHAX	−0.04	8.1 × 10^−1^
	ENSG00000082898	XPO1 (CRM1)	−0.16	4.6 × 10^−2^
Formation of a tertiary complex with SNU13	ENSG00000100697	DICER1	0.62	1.9 × 10^−9^
	ENSG00000113360	DROSHA	−1.45	1.7 × 10^−24^

## Data Availability

RNA-seq data have been deposited in the ArrayExpress database at EMBL-EBI (https://www.ebi.ac.uk/biostudies/arrayexpress, accessed on 1 August 2022) under accession number E-MTAB-12088 (small RNA fraction) and E-MTAB-9511 (poly(A)+ RNA fraction).

## Supplementary Materials

The supporting information can be downloaded at: https://www.mdpi.com/article/10.3390/ijms232213666/s1.
