# Supplementary material for: Analysis of Expression Pattern of snoRNAs in Human Cells A549 Infected by Influenza A Virus
Source: Int J Mol Sci. 2022 Nov 8;23(22):13666. doi: 10.3390/ijms232213666 (PMC9696202; doi:10.3390/ijms232213666)
Supplement: Supplementary file 1 [file ijms-23-13666-s001.zip › ijms-1891990-supplementary.pdf]

Table S1. Oligonucleotide primers for RT-qPCR.

RT-qPCR

Target	Name	Sequence (5' – 3')
U6 snRNA	U6_snRNA_for<br>U6_snRNA_rev	TCGCTTCGGCAGCACATATACTAAAAT<br>GAATTTGCGTGTCATCCTTGCG
U11 snRNA	U11_snRNA_for<br>U11_snRNA_rev	AAGGCTTCTGTCGTGAGTGGCACA<br>GGCGCCGGGACCAACGA
SNORD42A	F_sno42a<br>R_sno42a	CACGCAGGCTAATGATGG<br>CTTCAGTGGTTCCTTTGTTCATGTC
SNORD58A	F_sno58a<br>R_sno58a	GCAGTGATGACTTTCTTAGGAC<br>GCTGCTCAGAATTTATTAATTTTCACGGT
SNORD79	U79-f<br>U79-r	CTGTTAGTGATGATTTAA<br>CTGTTTCAGTTTAAGATT

stem-loop RT-qPCR

Target	Name	Sequence (5' – 3')
All first strand cDNA	SL_R1	GTGCAGGGTCCGAGGT
SNORD1B and sdRNAs-1B	F_sno1b_5_mir<br>R_SL_sno1b_5_mir<br>F_sno1b_3_mir<br>R_SL_sno1b_3_mir	CTGAGTCCATGATGATTTCAAG<br>GTCGTATCCAGTGCAGGGTCCGAGGTATTCGCACTGGATACGACCTTCAGAC<br>CACGCATTTCTGTGTGG<br>GTCGTATCCAGTGCAGGGTCCGAGGTATTCGCACTGGATACGACTTTCAGAT
SNORD8 and sdRNAs-8	F_sno8_5_mir<br>R_SL_sno8_5_mir<br>R_sno8	TCCCAATGATGAGTTGCCAT<br>GTCGTATCCAGTGCAGGGTCCGAGGTATTCGCACTGGATACGACGCTCAGT<br>CCCTCAGATCTTCATGTGAG

SNORD11 and sdRNAs-11	F_sno11_5_mir<br>R_SL_sno11_5_mir<br>F_sno11_3_mir<br>R_SL_sno11_3_mir	CACGCAGTTCAATGATGATTTC<br>GTCGTATCCAGTGCAGGGTCCGAGGTATTCGCACTGGATACGACAATCAGG<br>CACCAGGGCATCTTTAGTCAC<br>GTCGTATCCAGTGCAGGGTCCGAGGTATTCGCACTGGATACGACTGTCTCA
SNORD93B and sdRNAs-93B	F_sno93_5_mir<br>R_SL_sno93_5_mir<br>F_sno93_3_mir<br>R_SL_sno93_3_mir	GCATGGCCAAGGATGAGAAC<br>GTCGTATCCAGTGCAGGGTCCGAGGTATTCGCACTGGATACGACAATCAGA<br>CACCGATCTGCTGTGATGG<br>GTCGTATCCAGTGCAGGGTCCGAGGTATTCGCACTGGATACGACTGGCCTC

2′-O-methylation-specific RT-qPCR

Target	Name	Sequence (5′ – 3′)
28S:Am3809 (SNORD79)	Am_3809_F<br>Am_3809_R<br>Am_3809_In	GCGCATGAATGGATGAACG<br>CTGGATAGTAGGTAGGGACAG<br>ATGAATGGATGAACGAGATTCC
28S:Gm4198 (SNORD58a)	Gm_4198_F<br>Gm_4198_R<br>Gm_4198_In	GTCAAACGGTAACGCAGGT<br>GAGGTTTCTGTCCTCCCTG<br>GTAACGCAGGTGTCCTAAGG
18S:Am576 (SNORD93)	Am_576_F<br>Am_576_R<br>Am_576_In	GACTCTTTCGAGGCCCTGT<br>GCCCTCCAATGGATCCTC<br>GGAATGAGTCCACTTTAAATCC
28S:Gm4362 (SNORD1b)	Gm_4362_F<br>Gm_4362_R<br>Gm_4362_In	GGTTTTAAGCAGGAGGTGTC<br>CGTCGCTATGAACGCTTG<br>AGTTACCACAGGGATAACTGG

Table S2. Sequencing statistics of RNA-Seq. Samples Sm_A549_0h, Sm_A549_24h, Sm_A549_48h and pA_A549_0h, pA_A549_24h, pA_A549_48h include two biological replicates of small RNA and poly(A)+ RNA libraries respectively, of each time point (non-infected (0 h) and influenza-A/Puerto Rico/8/1934-infected (24 h and 48 h) A549 cells). Numbers of retrieved raw reads, reads after filtering (trimming of adapters, filtering by quality, removal of ribosomal RNA fragments) and uniquely mapped reads to GRCh38 and to Influenza A/Puerto Rico/8/1934 genomes are presented in table.

Small RNA-Seq

Sample	Biological repeat	Total read number	After filtering reads	Uniquely mapped reads	
				to hg38	to Influenza A
Sm_A549_0h	Sm_0h_1	5 876 508	4 545 646	3 772 700	10
	Sm_0h_2	6 455 310	5 254 025	4 371 753	7
Sm_A549_24h	Sm_24h_1	6 875 150	5 463 196	4 355 639	59 786
	Sm_24h_2	6 405 753	5 092 667	3 943 561	57 272
Sm_A549_48h	Sm_48h_1	6 551 298	4 956 558	3 246 757	69 033
	Sm_48h_2	6 095 045	4 509 835	2 957 440	63 165

polyA-selected RNA-Seq

Sample	Biological repeat	Total read number	After filtering reads	Uniquely mapped reads	
				to hg38	to Influenza A
pA_A549_0h	pA_0h_1	12 693 199	12 469 797	11 500 381	35
	pA_0h_2	10 610 878	10 411 687	9 598 270	323
pA_A549_24h	pA_24h_1	11 457 333	11 015 053	9 102 079	994 175
	pA_24h_2	11 097 112	10 621 125	8 547 535	1 139 113
pA_A549_48h	pA_48h_1	11 413 406	10 928 105	7 701 098	2 443 654
	pA_48h_2	11 604 763	11 135 881	7 732 196	2 597 907

Table S3. Differential expressed snoRNAs 24 h and 48 h after influenza A virus infection were identified using R package DESeq2 (v1.30.1) with a FDR-adjusted p -value < 0.05 and the absolute value of a log2(FC) > 0.58.

Differential expressed snoRNAs 24 h after influenza A virus infection

Up				Down			
**Ensembl ID**	**Gene symbol**	**log2(FC)**	***p*.adj**	**Ensembl ID**	**Gene symbol**	**log2(FC)**	***p*.adj**
ENSG00000221740	SNORD93	4.30	<< 0.05	ENSG00000206602	SNORD58A	-1.73	3.7E-30
ENSG00000207297	SNORD7	2.57	3.0E-28	ENSG00000252542	SNORD36C	-1.39	5.2E-07
ENSG00000201754	SNORD52	1.93	1.3E-49	ENSG00000271982	SNORD58B	-1.33	3.0E-22
ENSG00000199961	SNORD1B	1.48	1.1E-46	ENSG00000200608	SNORD114-11	-1.28	3.3E-05
ENSG00000200463	SNORD118	1.47	2.8E-27	ENSG00000207405	SNORA64	-1.13	3.4E-10
ENSG00000200785	SNORD8	1.08	8.6E-04	ENSG00000206597	SNORA57	-1.01	4.0E-05
ENSG00000277184	SNORA9	1.07	8.9E-03	ENSG00000202093	SNORD58C	-0.94	4.1E-15
ENSG00000212443	SNORA53	0.95	2.8E-02	ENSG00000221491	SNORA2C	-0.92	9.4E-03
ENSG00000249020	SNORA58	0.88	8.5E-03	ENSG00000264294	SNORD55	-0.83	1.6E-10
ENSG00000275662	SNORD112	0.82	2.4E-02	ENSG00000207279	SNORD116-24	-0.78	3.9E-02
ENSG00000199631	SNORD33	0.80	2.8E-08	ENSG00000264346	SNORA77B	-0.76	4.0E-02
ENSG00000207280	SNORD20	0.78	2.1E-06	ENSG00000207145	SNORA18	-0.73	3.3E-05
ENSG00000238622	SNORD97	0.77	2.6E-02	ENSG00000238649	SNORD42A	-0.69	1.4E-02
ENSG00000206620	SNORD45C	0.73	3.7E-03	ENSG00000277512	SNORD65	-0.64	1.9E-06
ENSG00000212158	SNORD66	0.66	9.4E-06	ENSG00000229686	SNORD56	-0.62	1.2E-04
ENSG00000238317	SNORD11	0.66	4.9E-03	ENSG00000238597	SNORD4B	-0.62	2.7E-05
ENSG00000221514	SNORD111B	0.65	6.6E-05	ENSG00000221066	SNORD111	-0.6	4.9E-04
ENSG00000202503	SNORD34	0.62	4.8E-03	ENSG00000200913	SNORD46	-0.59	7.3E-05

Differential expressed snoRNAs 48 h after influenza A virus infection

Up				Down			
**Ensembl ID**	**Gene symbol**	**log2(FC)**	***p*.adj**	**Ensembl ID**	**Gene symbol**	**log2(FC)**	***p*.adj**
ENSG00000277184	SNORA9	5.66	1.5E-57	ENSG00000206602	SNORD58A	-3.52	2.4E-118
ENSG00000221740	SNORD93	4.65	<< 0.05	ENSG00000200608	SNORD114-11	-2.91	1.0E-16
ENSG00000239183	SNORA84	4.53	1.5E-08	ENSG00000207001	SNORD116-2	-2.74	2.2E-04
ENSG00000207297	SNORD7	3.38	1.3E-49	ENSG00000221491	SNORA2C	-2.72	9.7E-15
ENSG00000238317	SNORD11	3.24	5.9E-58	ENSG00000271982	SNORD58B	-2.65	5.4E-82
ENSG00000201772	SNORA5C	3.23	1.3E-09	ENSG00000252542	SNORD36C	-2.55	9.6E-18
ENSG00000200463	SNORD118	2.96	1.2E-112	ENSG00000238961	SNORA47	-2.36	1.3E-07
ENSG00000238363	SNORA13	2.90	1.8E-11	ENSG00000207145	SNORA18	-2.13	1.3E-36
ENSG00000199785	SNORA52	2.89	5.2E-05	ENSG00000238917	SNORD10	-2.10	4.7E-08
ENSG00000200983	SNORA3A	2.87	2.9E-42	ENSG00000238649	SNORD42A	-2.06	9.0E-15
ENSG00000207067	SNORA72	2.81	1.3E-08	ENSG00000202093	SNORD58C	-2.01	5.8E-65
ENSG00000249020	SNORA58	2.74	2.8E-19	ENSG00000206597	SNORA57	-1.96	2.4E-15
ENSG00000202363	SNORA62	2.66	5.2E-35	ENSG00000207405	SNORA64	-1.96	1.3E-26
ENSG00000201129	SNORA58B	2.58	2.4E-08	ENSG00000238597	SNORD4B	-1.93	4.6E-41
ENSG00000206622	SNORA69	2.57	1.3E-05	ENSG00000207166	SNORA68	-1.90	5.4E-06
ENSG00000209582	SNORA48	2.37	6.5E-06	ENSG00000264294	SNORD55	-1.89	1.1E-53
ENSG00000200418	SNORA63B	2.25	1.6E-03	ENSG00000221066	SNORD111	-1.77	6.0E-27
ENSG00000200785	SNORD8	2.20	3.4E-13	ENSG00000207496	SNORA7A	-1.75	4.0E-06
ENSG00000238622	SNORD97	2.18	6.3E-12	ENSG00000229686	SNORD56	-1.70	5.6E-28
ENSG00000199961	SNORD1B	2.17	1.3E-99	ENSG00000207088	SNORA7B	-1.62	2.3E-03
ENSG00000207304	SNORA8	2.17	9.4E-06	ENSG00000207279	SNORD116-24	-1.57	9.3E-05
ENSG00000221420	SNORA81	2.16	7.8E-07	ENSG00000277512	SNORD65	-1.54	9.2E-34
ENSG00000235408	SNORA71B	2.12	7.3E-05	ENSG00000276788	SNORD26	-1.53	2.3E-21
ENSG00000199593	SNORD114-14	2.04	1.0E-03	ENSG00000200913	SNORD46	-1.51	1.2E-26
ENSG00000207445	SNORD15B	2.02	3.8E-08	ENSG00000207063	SNORD116-1	-1.49	5.7E-03

ENSG00000277194	SNORD22	2.01	5.0E-11	ENSG00000200480	SNORD114-28	-1.38	1.2E-02
ENSG00000199293	SNORA21	1.85	3.0E-14	ENSG00000274582	SNORA16A	-1.38	4.3E-02
ENSG00000206885	SNORA75	1.83	2.0E-08	ENSG00000280498	SNORA16A	-1.38	4.3E-02
ENSG00000274998	SNORA17A	1.82	1.8E-02	ENSG00000254341	SNORD87	-1.31	9.4E-13
ENSG00000207280	SNORD20	1.67	2.4E-27	ENSG00000221539	SNORD99	-1.28	4.2E-32
ENSG00000212464	SNORA12	1.67	4.2E-02	ENSG00000275043	SNORD25	-1.28	1.2E-26
ENSG00000206620	SNORD45C	1.63	1.6E-12	ENSG00000200406	SNORD114-23	-1.19	1.4E-03
ENSG00000206941	SNORD15A	1.63	2.5E-47	ENSG00000200623	SNORD18A	-1.17	8.9E-19
ENSG00000207493	SNORA46	1.62	7.6E-05	ENSG00000207093	SNORD116-8	-1.16	2.5E-07
ENSG00000212135	SNORD67	1.61	5.2E-04	ENSG00000199744	SNORD36A	-1.12	4.0E-05
ENSG00000274091	SNORD1C	1.55	1.1E-20	ENSG00000199575	SNORD114-1	-1.09	1.3E-03
ENSG00000202270	SNORD114-12	1.50	1.4E-05	ENSG00000274544	SNORD28	-1.06	2.7E-04
ENSG00000201754	SNORD52	1.48	7.9E-30	ENSG00000207442	SNORD116-6	-1.04	4.3E-02
ENSG00000202503	SNORD34	1.45	3.7E-13	ENSG00000238344	SNORD126	-0.99	1.4E-12
ENSG00000212232	SNORD17	1.45	8.9E-29	ENSG00000201823	SNORD48	-0.94	1.5E-17
ENSG00000275084	SNORD91B	1.33	5.3E-03	ENSG00000264346	SNORA77B	-0.91	1.6E-02
ENSG00000199631	SNORD33	1.32	1.9E-21	ENSG00000209482	SNORD83A	-0.88	4.5E-07
ENSG00000275662	SNORD112	1.25	4.5E-04	ENSG00000207031	SNORD59A	-0.81	3.2E-11
ENSG00000201240	SNORD114-9	1.21	9.7E-08	ENSG00000212304	SNORD12	-0.80	2.0E-12
ENSG00000200354	SNORA71D	1.18	5.2E-04	ENSG00000221381	SNORD88B	-0.78	2.6E-05
ENSG00000272533	SNORA28	1.17	4.4E-02	ENSG00000207392	SNORA20	-0.77	6.4E-04
ENSG00000201302	SNORA65	1.15	3.3E-03	ENSG00000209702	SNORD41	-0.77	3.3E-02
ENSG00000265145	SNORD53	1.12	2.6E-03	ENSG00000206680	SNORD21	-0.75	9.9E-11
ENSG00000201998	SNORA23	1.11	1.1E-02	ENSG00000209042	SNORD12C	-0.70	8.1E-09
ENSG00000200087	SNORA73B	1.09	7.3E-03	ENSG00000212607	SNORA3B	-0.69	1.9E-02
ENSG00000278274	SNORA61	1.08	4.0E-06	ENSG00000277846	SNORD30	-0.68	4.6E-08
ENSG00000207475	SNORA80E	1.06	4.0E-02	ENSG00000238531	SNORD105B	-0.67	3.8E-04
ENSG00000207375	SNORD116-23	1.03	3.0E-02	ENSG00000208797	SNORD73A	-0.66	2.0E-02
ENSG00000200831	SNORD36B	1.01	2.6E-13	ENSG00000212309	SNORD70B	-0.64	5.5E-03

ENSG00000212158	SNORD66	0.97	1.2E-11
ENSG00000277887	SNORA50C	0.95	2.6E-03
ENSG00000272344	SNORD114-21	0.94	1.2E-02
ENSG00000207118	SNORD14D	0.92	5.3E-08
ENSG00000212447	SNORD90	0.91	2.1E-06
ENSG00000208772	SNORD94	0.77	6.5E-03
ENSG00000200879	SNORD14E	0.75	9.7E-03
ENSG00000221116	SNORD110	0.74	9.7E-03
ENSG00000221514	SNORD111B	0.69	2.0E-05
ENSG00000265236	SNORD84	0.69	4.4E-04
ENSG00000207421	SNORD38B	0.68	1.4E-05
ENSG00000281859	SNORD38B	0.68	1.4E-05
ENSG00000239039	SNORD13	0.63	7.5E-05

ENSG00000212452	SNORD69	-0.61	3.9E-06
ENSG00000202314	SNORD6	-0.60	3.3E-06

Table S4. Differential expressed mature miRNAs 24 h and 48 h after influenza A virus infection were identified using R package DESeq2 (v1.30.1) with the baseMean > 10, FDR-adjusted p -value < 0.05 and the absolute value of a log2(FC) > 0.58.

Differential expressed mature miRNAs 24 h after influenza A virus infection

Up				Down			
**miRBase ID**	**baseMean**	**log2(FC)**	***p*.adj**	**miRBase ID**	**baseMean**	**log2(FC)**	***p*.adj**
hsa-miR-146a-5p	27	4.69	1.8E-05	hsa-miR-138-1-3p	14	-2.66	4.8E-03
hsa-miR-190b-5p	43	4.14	3.3E-10	hsa-miR-194-3p	18	-2.64	1.2E-03
hsa-miR-215-5p	157	3.65	2.4E-28	hsa-miR-27b-5p	887	-2.27	5.0E-64
hsa-miR-200c-3p	723	2.66	2.6E-71	hsa-miR-365a-5p	56	-2.06	5.3E-07
hsa-miR-449c-5p	45	2.36	9.0E-06	hsa-miR-4521	38	-1.95	9.2E-05
hsa-miR-146b-5p	1060	1.44	3.3E-27	hsa-miR-125b-1-3p	80	-1.58	7.1E-06
hsa-miR-139-5p	37	1.43	2.0E-02	hsa-miR-23b-3p	2628	-1.00	1.9E-13
hsa-miR-450a-5p	114	1.26	4.8E-05	hsa-miR-25-5p	96	-0.88	1.3E-02
hsa-miR-212-5p	47	1.07	4.2E-02	hsa-miR-424-3p	180	-0.81	1.0E-03
hsa-miR-615-3p	58	1.05	3.0E-02	hsa-miR-370-3p	564	-0.78	7.6E-06
hsa-miR-132-5p	75	1.01	1.3E-02	hsa-miR-941	433	-0.78	3.0E-05
hsa-miR-450b-5p	262	0.86	4.8E-05	hsa-miR-222-3p	11440	-0.69	1.6E-06
hsa-miR-148a-3p	1148	0.81	7.3E-08	hsa-miR-744-5p	422	-0.66	2.1E-04
hsa-miR-196b-5p	337	0.75	9.2E-05	hsa-miR-23a-3p	516	-0.62	1.5E-04
hsa-miR-10b-5p	3827	0.71	9.8E-11	hsa-miR-224-5p	9543	-0.60	1.1E-07
hsa-miR-181a-5p	947	0.67	5.0E-06	hsa-miR-1307-3p	2106	-0.59	1.1E-06
hsa-miR-96-5p	897	0.62	2.1E-05				

Differential expressed mature miRNAs 48 h after influenza A virus infection

Up				Down			
**miRBase ID**	**baseMean**	**log2(FC)**	***p*.adj**	**miRBase ID**	**baseMean**	**log2(FC)**	***p*.adj**
hsa-miR-146a-5p	27	5.04	2.0E-06	hsa-miR-138-1-3p	14	-3.54	7.9E-04
hsa-miR-215-5p	157	3.82	4.4E-31	hsa-miR-29b-1-5p	15	-3.33	1.1E-03
hsa-miR-26a-2-3p	14	3.73	1.4E-04	hsa-miR-4521	38	-3.16	2.8E-08
hsa-miR-190b-5p	43	3.72	1.7E-08	hsa-miR-365b-5p	11	-2.97	9.9E-03
hsa-miR-200c-3p	723	2.61	7.8E-68	hsa-miR-194-3p	18	-2.70	1.4E-03
hsa-miR-449c-5p	45	2.19	4.3E-05	hsa-miR-365a-5p	56	-2.60	2.3E-09
hsa-miR-146b-5p	1060	1.60	1.9E-33	hsa-miR-27b-5p	887	-2.55	7.1E-75
hsa-miR-139-5p	37	1.51	1.0E-02	hsa-miR-25-5p	96	-2.39	1.0E-11
hsa-miR-181a-5p	947	1.45	3.0E-27	hsa-miR-539-5p	19	-2.10	1.6E-02
hsa-miR-132-5p	75	1.37	2.0E-04	hsa-miR-195-3p	32	-2.05	6.6E-04
hsa-miR-19b-3p	132	1.32	1.2E-06	hsa-miR-92a-1-5p	67	-2.00	2.8E-06
hsa-miR-450a-5p	114	1.23	8.2E-05	hsa-miR-485-3p	38	-1.94	1.1E-03
hsa-miR-615-3p	58	1.21	8.4E-03	hsa-miR-125b-1-3p	80	-1.88	1.3E-07
hsa-miR-193b-3p	93	1.20	3.7E-04	hsa-miR-27a-5p	617	-1.75	4.6E-29
hsa-miR-454-3p	49	1.17	2.2E-02	hsa-miR-92b-5p	67	-1.68	5.4E-05
hsa-miR-212-5p	47	1.07	4.0E-02	hsa-miR-3662	28	-1.67	1.6E-02
hsa-miR-29a-3p	2601	1.00	5.3E-14	hsa-miR-320b	20	-1.66	4.8E-02
hsa-miR-181b-5p	603	0.98	1.1E-09	hsa-miR-424-3p	180	-1.66	1.0E-11
hsa-miR-148a-3p	1148	0.90	7.6E-10	hsa-miR-432-5p	40	-1.37	1.2E-02
hsa-miR-186-5p	927	0.90	2.9E-11	hsa-let-7c-5p	855	-1.36	2.0E-16
hsa-miR-29b-3p	133	0.89	1.5E-03	hsa-miR-7974	353	-1.33	6.7E-12
hsa-miR-181d-5p	230	0.73	1.2E-03	hsa-let-7b-5p	6765	-1.33	5.8E-27
hsa-miR-450b-5p	262	0.68	2.1E-03	hsa-miR-224-5p	9543	-1.28	6.5E-34
hsa-miR-10b-5p	3827	0.68	5.3E-10	hsa-miR-23b-3p	2628	-1.07	2.2E-15
hsa-miR-221-3p	1958	0.68	4.2E-06	hsa-miR-222-3p	11440	-1.02	9.1E-14

hsa-miR-30d-5p	19433	0.64	1.1E-10	hsa-miR-370-3p	564	-0.99	5.9E-09
hsa-miR-191-5p	3746	0.64	8.7E-09	hsa-miR-1304-5p	68	-0.98	1.8E-02
hsa-miR-194-5p	2096	0.60	2.8E-06	hsa-miR-1180-3p	687	-0.97	4.0E-09
hsa-miR-32-5p	576	0.60	2.9E-03	hsa-miR-1307-3p	2106	-0.95	1.4E-16
hsa-miR-26a-5p	31364	0.59	5.0E-09	hsa-miR-330-3p	251	-0.95	6.9E-06
				hsa-miR-320a-3p	4482	-0.95	3.0E-17
				hsa-miR-30c-2-3p	109	-0.80	1.5E-02
				hsa-miR-941	433	-0.77	3.3E-05
				hsa-miR-134-5p	100	-0.75	3.9E-02
				hsa-miR-425-5p	888	-0.68	1.3E-06
				hsa-miR-409-3p	1184	-0.64	1.1E-04

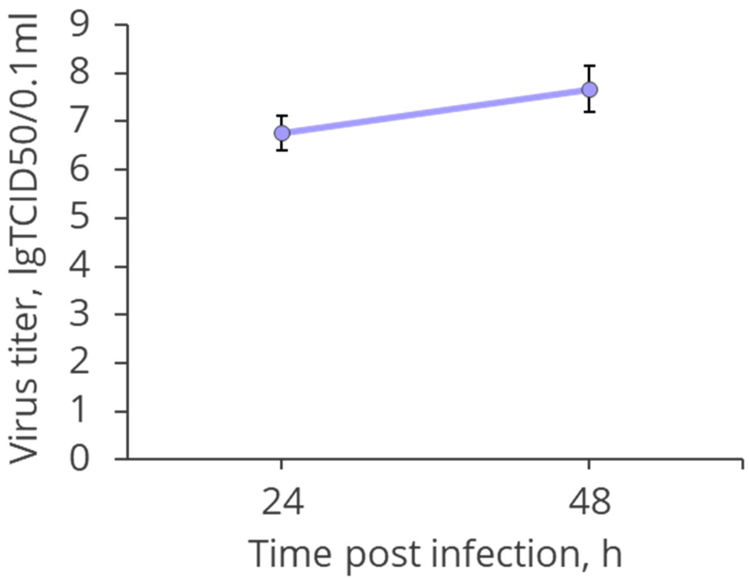

Figure S1. Growth kinetics of influenza A/Puerto Rico/8/1934 (H1N1) virus in A549 cell line. At the indicated time points post infection, virus titers were determined by use of plaque assays in MDCK cells. Values shown are the means (± s.d.) of three independent experiments.

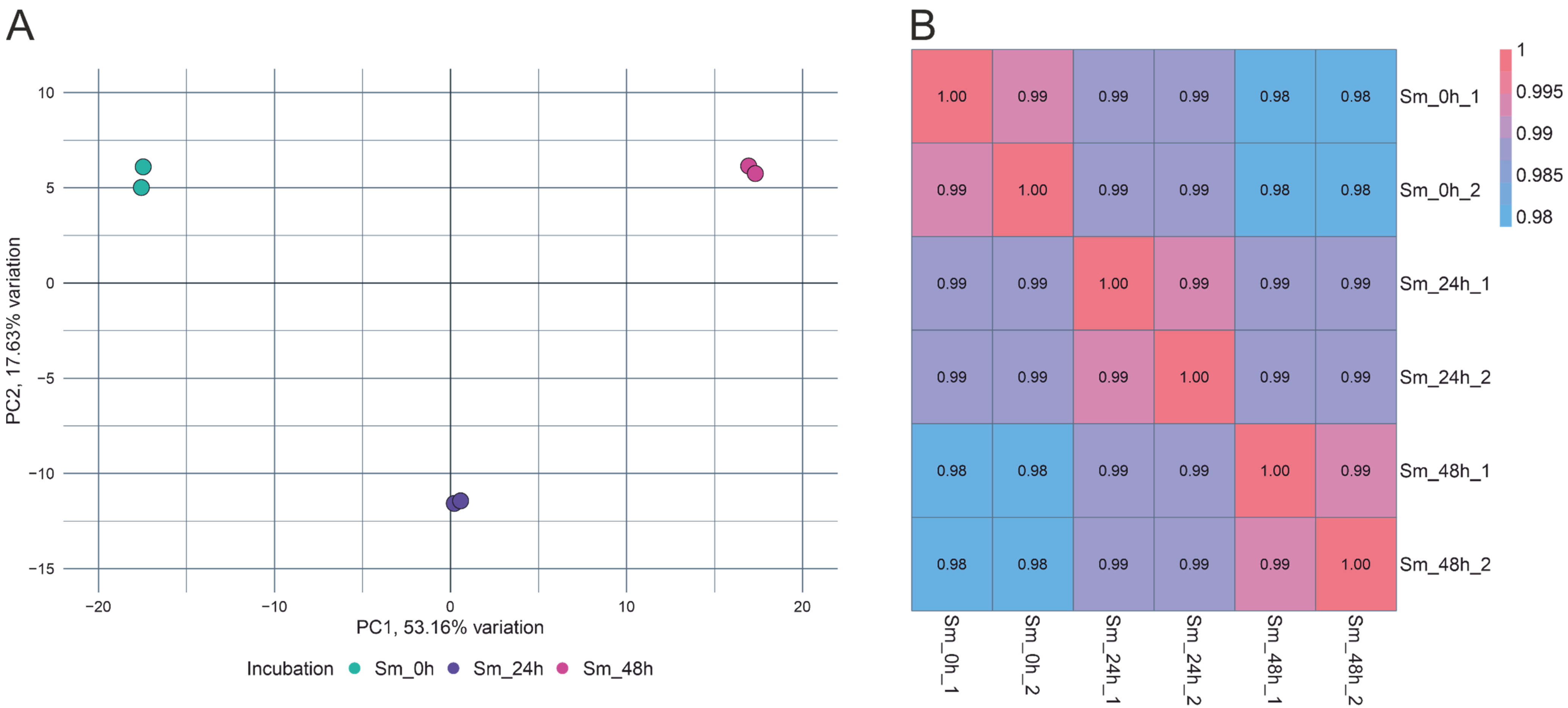

Figure S2. Differential gene expression analysis of 6 cDNA libraries for small RNA fraction. **A.** Principal component analysis (PCA). Each dot represents one biological repeat. **B.** Heatmap plots showing sample-to-sample correlations.

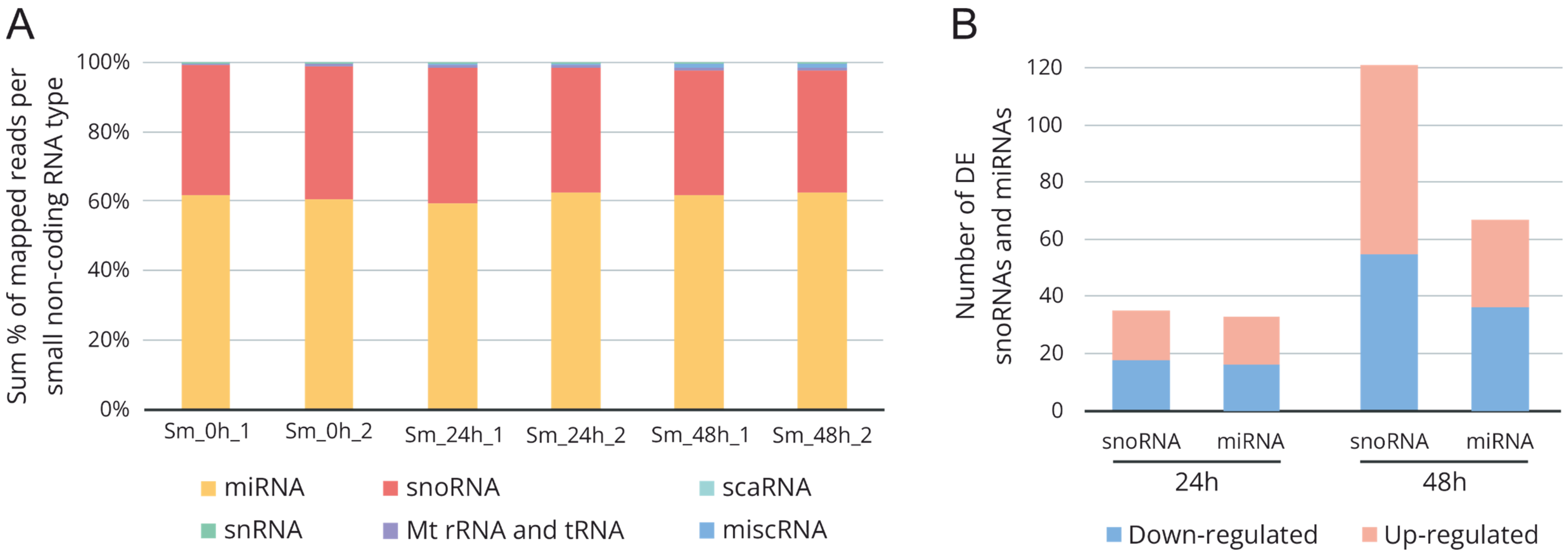

Figure S3. A. Relative abundance of different small non-coding RNA types (miRNA, snoRNA, scaRNA, snRNA, Mt rRNA and tRNA, miscRNA) per small RNA library. **B.** Diagrams show the number of up-regulated and down-regulated (differentially expressed (DE)) snoRNAs and miRNAs 24 h and 48 h after influenza A virus infection (FDR-adjusted p-value < 0.05, absolute value of a log2 (FC) > 0.58).

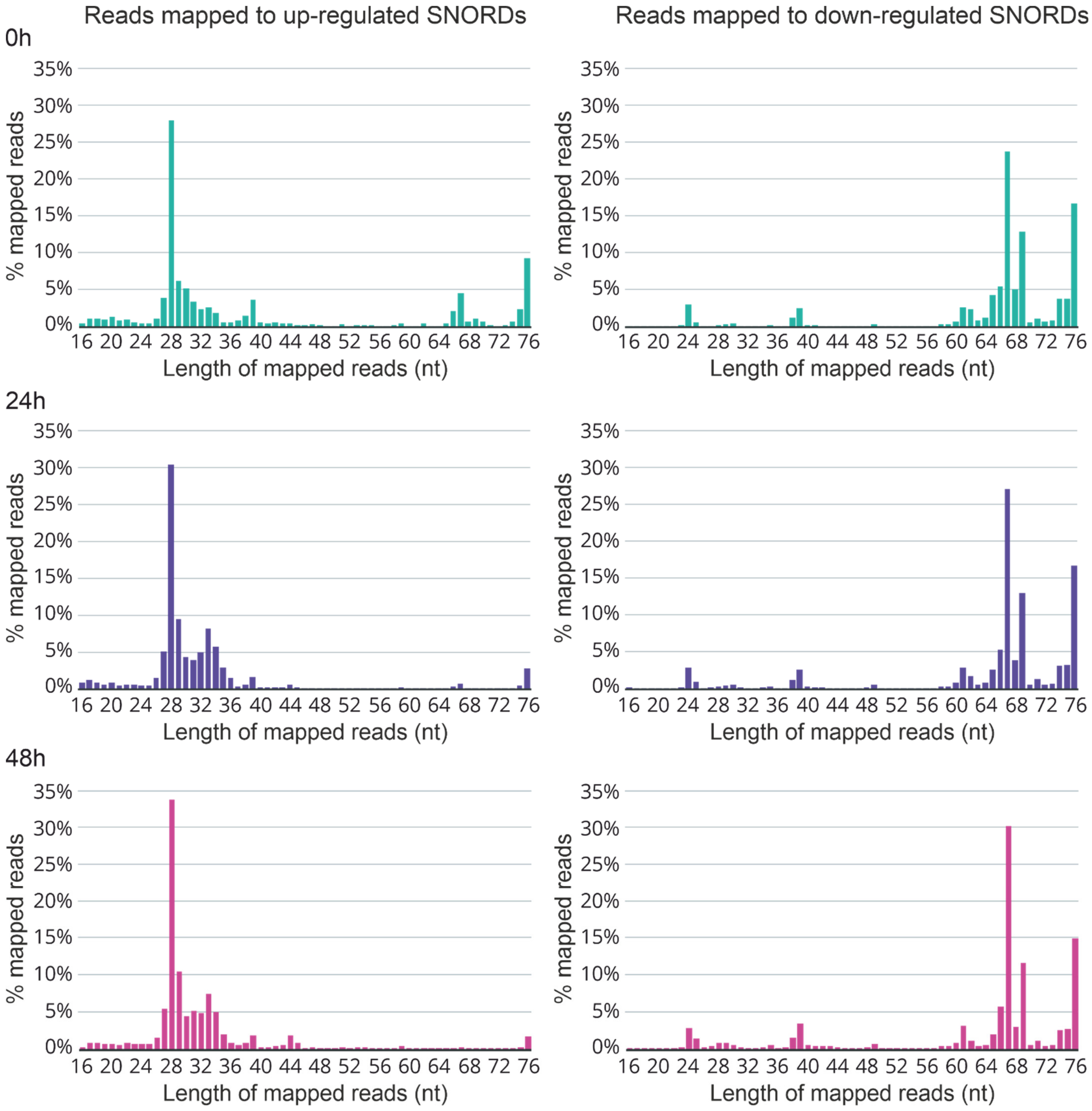

Figure S4. Size distribution of reads mapped to up-regulated and down-regulated C/D-box snoRNAs (SNORDs) in infected A549 cells after 0 h (non-infected), 24 h and 48 h incubation.

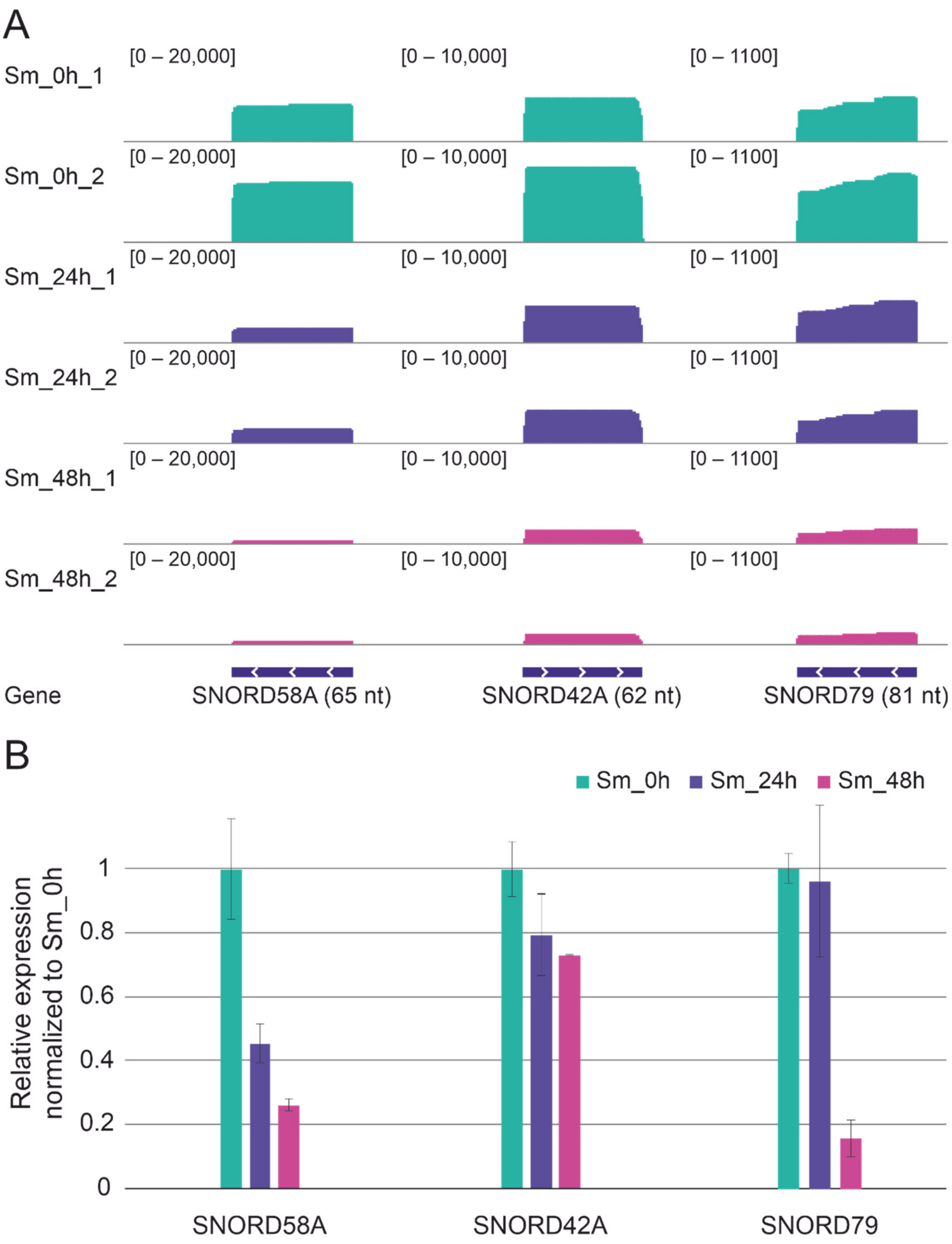

Figure S5. A. The coverage tracks of the aligned reads for the three small nucleolar RNAs down-regulated in response to influenza A virus infection (SNORD58A, SNORD42A and SNORD79) generated with IGV. Green reads, non-infected cells; violet reads, infected cells after 24 h incubation; pink reads, infected cells after 48 h incubation. **B**. Expression of full length SNORD58A, SNORD42A and SNORD79 measured by quantitative RT-qPCR.
